# Anti-Inflammatory and Antidiarrheal Effects of Two Strains of Lactic Acid Bacteria Isolated from Healthy Pets on *Escherichia coli* K88-Induced Diarrhea in Mice

**DOI:** 10.3390/microorganisms13020239

**Published:** 2025-01-22

**Authors:** Ya Zhao, Shukun Liang, Xiaoxin Fu, Yaping Guo, Yu Wang, Jiaxue Wang, Xiumin Wang, Zhenlong Wang, Hui Tao, Bing Han, Jinquan Wang

**Affiliations:** 1 Key Laboratory of Feed Biotechnology of Ministry of Agriculture and Rural Affairs, Institute of Feed Research, Chinese Academy of Agricultural Sciences, Beijing 100081, China; 82101225430@caas.cn (Y.Z.); 18811536925@163.com (S.L.); fuxiaoxin2000@outlook.com (X.F.); guoyaping@nwafu.edu.cn (Y.G.); wangyu_ivy@163.com (Y.W.); 19904762097@163.com (J.W.); wangxiumin@caas.cn (X.W.); wangzhenlong02@caas.cn (Z.W.); taohui@caas.cn (H.T.); 2 College of Veterinary Medicine, China Agricultural University, Beijing 100193, China

**Keywords:** *Lacticplantibacillus plantarum*, *LimosiLactobacillus reuteri*, diarrhea, 16S rDNA, inflammation, pathway

## Abstract

Lactic acid bacteria play a crucial role in maintaining the health of the host’s gut microbiota. In this study, the anti-inflammatory properties of *Limosilactobacillus reuteri* LR20-6 and *Lacticplantibacillus plantarum* L272 were evaluated using a mouse model of diarrhea induced by *Escherichia coli*. We also investigated their effects on gut microbiota regulation. The results indicated that both *Lacticplantibacillus plantarum* and *Limosilactobacillus reuteri* could reduce inflammation by inhibiting the expression of inflammatory factors IL-6 and TNF-α and blocking the MyD88 and NF-kB/p65 signaling pathways. Additionally, after intervention with these strains, the relative abundance of *Lactobacillus* was significantly increased. This suggested that *Lacticplantibacillus plantarum* and *Limosilactobacillus reuteri* could mitigate the severity of *E. coli*-induced diarrhea and enhance the abundance of beneficial probiotics in the gut of animals.

## 1. Introduction

As the aging of dogs and cats intensifies, the incidence of diarrhea is also rising. Diarrhea can trigger or exacerbate inflammatory bowel disease (IBD), which includes Crohn’s disease (CD) and ulcerative colitis (UC) [1]. IBD is relatively common in dogs, especially middle-aged and older animals. Clinically, treatment strategies can be divided into three categories: first, drug therapy, where two main types of medications are used to control inflammation. One category includes immunosuppressive drugs, such as corticosteroids and immunosuppressants, while the other includes antibiotics like metronidazole or enrofloxacin. Another strategy is to regulate the gut microbiota by supplementing with probiotics, which helps restore gut health. In cases of severe damage, such as localized intestinal lesions or severe ulcerative colitis, surgical intervention may be required for treatment. In drug therapy, overuse of antibiotics has led to an increase in antibiotic resistance, and excessive antibiotic residues present serious concerns [2]. Probiotics can improve the microbial composition, thereby preventing diarrhea and balancing the pet’s gut microbiota.

Recent studies have shown that yeast [3], *Bacillus subtilis* [4], *Lactobacillus species* [5], and *Bifidobacterium species* [6] can be used as probiotic materials, with Lactobacillus species considered the most promising. As a heterologous fermentation lactic acid bacterium, *LimosiLactobacillus reuteri* [7] has garnered widespread attention for its applications in pet gut health. The existing literature suggests that *LimosiLactobacillus reuteri* has antidiarrheal effects in animals such as mice, rats, and humans. Some researchers have isolated *LimosiLactobacillus reuteri* from the feces of breastfed healthy infants [8,9]. This strain has shown the best overall performance in five aspects: acid tolerance, tolerance to bile salts and intestinal fluids, surface hydrophobicity, bacterial self-aggregation, and strain adhesion. Feeding this strain to IBD mice increased the diversity of their gut microbiota, reduced pro-inflammatory pathways, enhanced anti-inflammatory and gut protective pathways, and improved IBD symptoms in the mice [10]. *Lactiplantibacillus plantarum* plays a crucial role in gastrointestinal health, a phenomenon that has been widely studied. The isolated *Lacticplantibacillus plantarum* and *LimosiLactobacillus reuteri* exhibited strong growth performance, antimicrobial activity, and adaptability to low pH [11,12]. In rats with irritable bowel syndrome (IBS), oral administration of *Lacticplantibacillus plantarum* strengthened the colonic barrier function [13]. *Lacticplantibacillus plantarum* has also been shown to alleviate symptoms associated with gastrointestinal diseases [14,15].

This study comprehensively evaluated *LimosiLactobacillus reuteri* LR20-6 and *Lacticplantibacillus plantarum* L272 from pet feces. This study aimed to investigate the effects of these two probiotics on *Escherichia coli (E. coli)*-induced diarrhea in mice, explore potential mechanisms, and assess their potential benefits for animal health. It also explored their role in treating diarrhea, laying the foundation for further application and development in future.

## 2. Materials and Methods

### 2.1. Test Strains and Preparation

*LimosiLactobacillus reuteri* 20-6 (CGMCC No. 27590) and *Lacticplantibacillus plantarum* L272 (CGMCC No. 27193) were isolated, identified, and preserved in our laboratory. *E. coli* K88 (ATCC) was purchased from China Microbial Species Preservation Management Committee.

### 2.2. Experimental Animals and Reagents

Forty healthy male Bal b/c mice, 6 weeks old, were provided by Beijing Vital River Laboratory Animal Technology Co., Ltd. (Beijing, China). During the experiment, they had ad libitum access to food and distilled water, with regular bedding changes. The temperature was maintained at 20–23 °C with a 12 h light/dark cycle.

The animal test was approved by the Laboratory Animal Ethical Committee and its inspection of the Institute of Feed Research of CAAS (IFR-CAAS-20230510).

### 2.3. Mouse Model

After 3 days of acclimation, Bal b/c mice were randomly divided into 5 groups, with 8 mice per group and 4 mice per cage. The groups included the following: group CK (with PBS, CK), group LR (with *LimosiLactobacillus reuteri* LR20-6, 1 × 10^11^ CFU per mouse), group L272 (*Lacticplantibacillus plantarum* L272, 1 × 10^11^ CFU per mouse), group Augmetin (with the antibiotic amoxicillin–clavulanate, 0.45 mg/kg), and group M (without any probiotics). After the 7-day period for adaptation, treatment was initiated through gavage. Mice in the LR, L272, Augmentin, and M groups were each gavaged daily with 200 µL of *E. coli* at a concentration of at least 1 × 10^13^ CFU/mL for 7 days. The CK group was gavaged daily with an equal volume of PBS for 7 days. After gavage for 7 days, the CK and M groups were gavaged with PBS, the LR and L272 groups were gavaged with probiotic preparations (with a dose of at least 1 × 10^11^ CFU per mouse), and the Augmentin group was gavaged with amoxicillin–clavulanate (0.45 mg/kg). The experiment lasted for 21 days. The initial body weight and food intake were tested for each group of mice. The diarrhea situation was tested every day.

Weight Gain Rate = (Final Weight − Initial Weight)/Initial Weight × 100%

Diarrhea Rate = (Number of Mice with Diarrhea/Total Number of Mice in the Group) × 100%

### 2.4. Intestinal Epithelial Barrier Function

Duodenal samples were collected from the mice and then fixed in a tissue fixation solution. The samples were processed and stained with Hematoxylin and Eosin by Beijing Huaying Biotechnology Research Institute (Beijing, China).

### 2.5. Anti-Inflammatory Cytokine

Blood was collected from the mice via ocular puncture, and was then centrifuged at 2000 rpm for 10 min at 4 °C. The serum levels of sIgA, IL-6, and TNF-α were measured using an enzyme-linked immunosorbent assay (ELISA) following the instructions in the ELISA kit (Jiangsu Meimian Industrial Co., Ltd., Yan Cheng, Jiangsu, China).

### 2.6. Western Blot

Mouse colon tissue was harvested and homogenized with lysis buffer. The protein concentrations of the samples were determined, and then 70 μg of total protein per sample was loaded onto an SDS-PAGE gel. The protein was transferred to a nitrocellulose membrane. Then, the membrane was blocked and incubated overnight at 4 °C with rabbit anti-MyD88 polyclonal antibody (1:1000), rabbit anti-NF-kB/p65 polyclonal antibody (1:1000), and mouse anti-β-Actin monoclonal antibody (1:1000). The membrane was washed with TBST buffer, and then incubated with horseradish peroxidase-conjugated secondary antibody (1:10,000) at room temperature. The membrane was washed again and the bands were visualized using the BIO-RAD gel imaging system.

### 2.7. Determination of the Mouse Fecal Microbiota

The E.Z.N.A. Mag-Bind Soil DNA Kit (Omega, M5635-02, Norcross, GA, USA) was used to extract microbial genomic DNA from 40 colon content samples. The DNA concentration of the samples was measured using the QuBit dsDNA HS assay. The extracted DNA samples were stored at −80 °C for PCR amplification. The PCR products were purified and quantified using gel extraction and detection kits. The extracted DNA samples were sent to Shenggong Biotechnology (Shanghai, China) for 16S rDNA sequencing.

### 2.8. Data Statistical Analysis

Data analysis was performed using GraphPad Prism 9.5.1 (San Diego, CA, USA) for one-way ANOVA to test for significant differences. Results are expressed as mean ± standard error of the mean (SEM). *p* < 0.05 was considered statistically significant. Graphs were created using GraphPad Prism 9.2. The 16S rDNA in the NCBI Sequence is SUB14835894.

## 3. Results

### 3.1. Growth Performance

**Figure 1:**
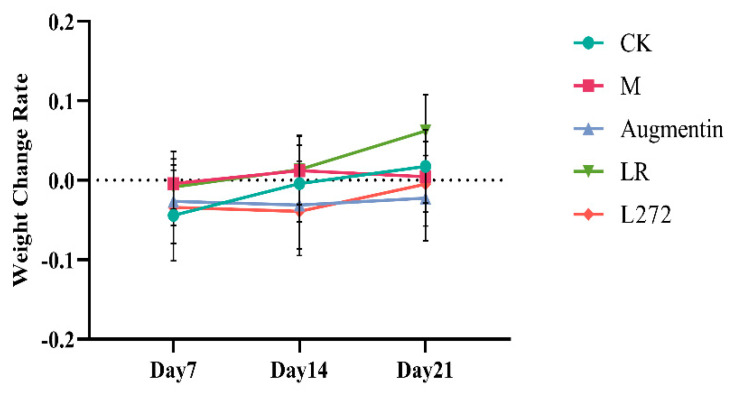
Change rate of mice body weight.

In the normal group, mice exhibited good health, glossy fur, normal bowel movements, and steady weight gain. In contrast, after modeling, the mice in all groups showed reduced appetite, lethargy, dull fur, loose stools with blood, and weight loss. Compared to the model group, mice in the LR and L272 treatment groups showed increased food intake, greater activity, more formed stools without blood, and gradual weight gain. As shown in Figure 1, the mice’s weight began to decline on the 7th day of *E. coli* gavage; after stopping *E. coli* gavage and treating for 7 days (on the 14th day), the weight gain rate started to rise. After 21 days of gavage, weight gain rates were higher in the normal and three treatment groups compared to the model group, with the most significant increase in the LR group, a notable increase in the L272 group, and a more moderate increase in the Augmentin group.

### 3.2. Diarrhea Index

As shown in Table 1, on the 7th day after *E. coli* gavage, all groups except the CK group had a higher diarrhea rate, with the diarrhea index in each group reaching its peak on the 7th day of modeling. By the 21st day, after three different treatments, the diarrhea index in the antibiotic intervention group had decreased significantly compared to the M group (*p* < 0.01). The diarrhea indices also decreased in the LR group and the L272 group.

### 3.3. Blood Inflammatory Factors

**Figure 2:**
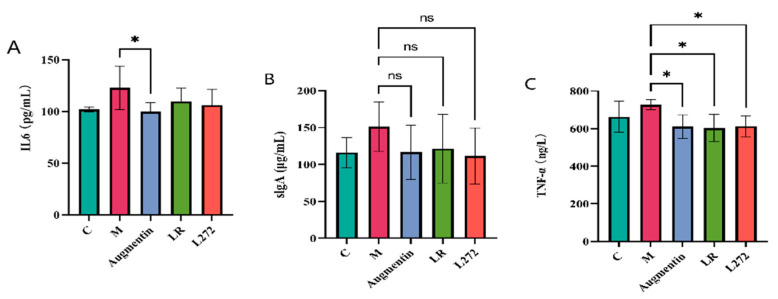
The impact on the expression of inflammatory cytokines (**A**) IL-6; (**B**) sIgA; and (**C**) TNF-α. Statistics were calculated with one-way ANOVA to test. ns, no significant difference; *, *p* < 0.05; data are presented as the mean ± SEM.

ELISA kits were used to measure the levels of secretory IgA (sIgA), IL-6, and TNF-α in the serum of mice from each group, as shown in Figure 2. After 14 days of treatment, compared to the CK group, the M group mice showed increased serum levels of IL-6, sIgA, and TNF-α, but the differences were not statistically significant. Compared to the M group, all three treated groups showed a decrease in inflammatory factors. Notably, the Augmentin group had a significant reduction in serum TNF-α and IL-6 levels (*p* < 0.05), while the LR and L272 groups had significant reductions in serum TNF-α levels (*p* < 0.05).

### 3.4. Histological Observation of Duodenal Tissue in Each Group

**Figure 3:**
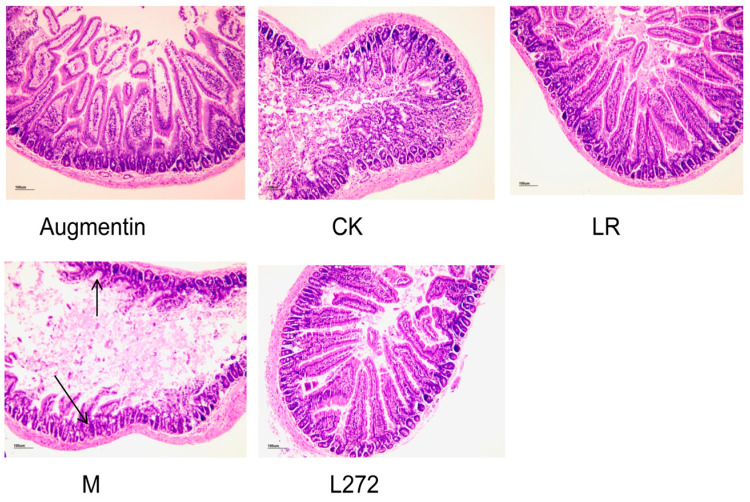
Representative images of H&E staining histology scores of colons. (×100) in the five groups—CK, LR, L272, Augmentin, and M—of mice.

As shown in Figure 3 the duodenal mucosa of the CK group mice showed no inflammatory damage. In the M group, the duodenal mucosa was mostly incomplete and exhibited inflammatory changes. There was significant loss of mucosal folds, a marked reduction in goblet cells, and pronounced infiltration of inflammatory cells in the lamina propria and submucosa. The villus height and crypt depth were the lowest in this group. Compared to the M group, the duodenal mucosa in the Augmentin, LR, and L272 groups showed more complete structures, reduced inflammatory cell infiltration, and increased villus length and crypt depth. The pathological scores, from highest to lowest, were as follows: M group > LR group > L272 group > Augmentin group > CK group; LR and L272 had significantly reduced histological scores compared to the M group (Figure 3).

### 3.5. Impact on Intestinal Epithelial Barrier Function

**Figure 4:**
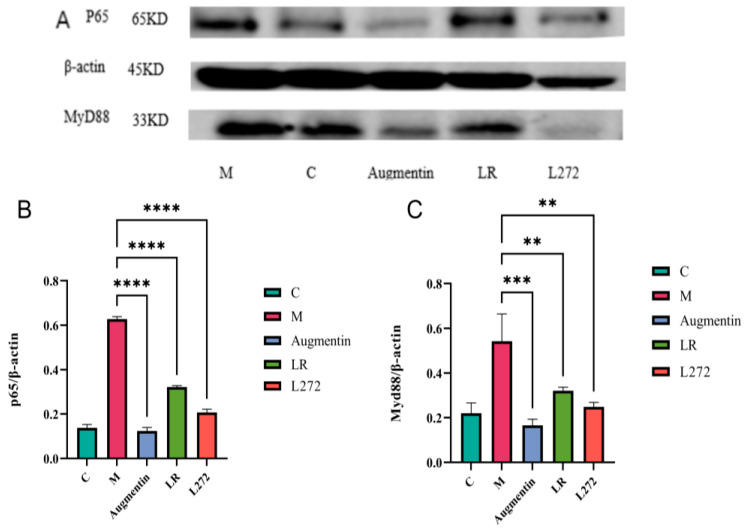
Western blot results for the colon tissues of mice in the five groups—CK, LR, L272, Augmentin, and M. (**A**) Mouse WB protein bands; (**B**) the expression levels of NF-kB p65; and (**C**) the expression levels of MyD88. Statistics were calculated with one-way ANOVA to test. **, *p* < 0.01; ***, *p* < 0.001; ****, *p* < 0.0001. Data are presented as the mean ± SEM.

As shown in Figure 4, compared to the normal group, the expression levels of MyD88 and NF-kB p65 signaling pathway proteins were significantly increased in the M group. In contrast, the expression levels of these proteins were significantly reduced in the colon tissues of the Augmentin, LR, and L272 groups compared to the M group (*p* < 0.01).

### 3.6. Microbiota Sequencing Results and Diversity Analysis

**Figure 5:**
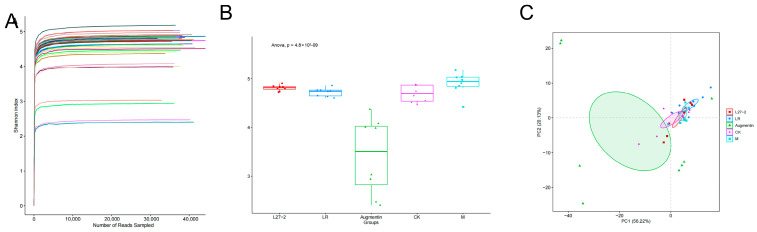
Alpha and Beta diversity analysis of presence of mice. (**A**) Shannon–Wiener curves for each group of mice; (**B**) alpha diversity indices for each group of mice; and (**C**) PCA for each group of mice.

**Figure 6:**
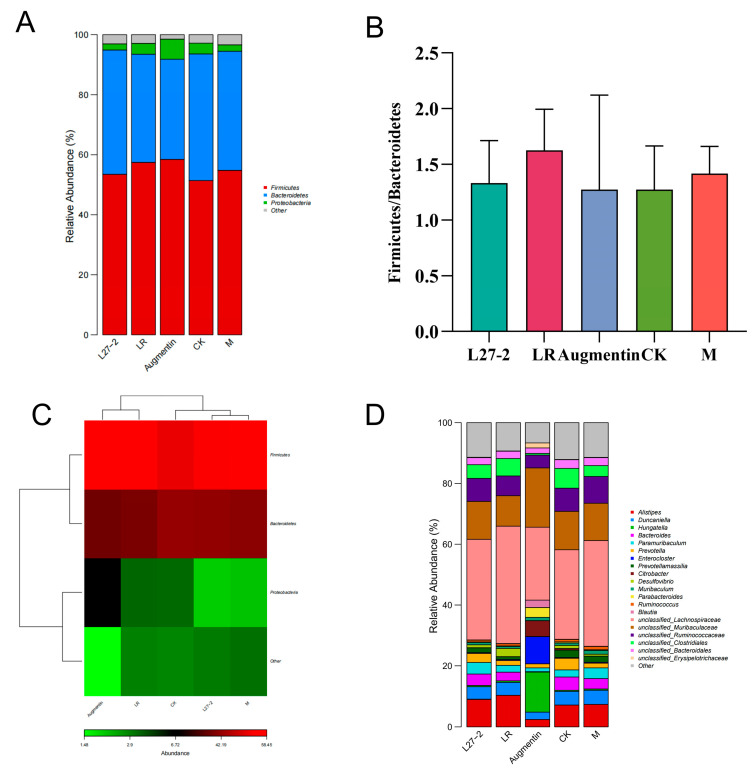
Analysis of the gut microbiota species composition in feces from different groups of rats. (**A**) Bar chart of species relative abundance at the phylum level; (**B**) ratio of Firmicutes to Bacteroidetes; (**C**) heatmap of dominant species at the phylum level; and (**D**) bar chart of species relative abundance at the genus level.

The Shannon–Wiener curves for the five groups—CK, LR, L272, Augmentin, and M—are shown in Figure 5. Each group consisted of eight samples. On the *x*-axis, we have the number of randomly sampled sequences, and on the *y*-axis, the corresponding alpha diversity index. As the number of sequences increases, the curves for each sample rise sharply and then level off, indicating that the sampling depth in this experiment was sufficient. The 16S rRNA sequencing depth was adequate, and the data volume was sufficient to reflect the microbial community characteristics in the samples, allowing for subsequent in-depth statistical and visual analyses. The alpha diversity indices (Chao1 and Shannon indices) of the fecal microbiomes from each group of mice were used to evaluate community richness and diversity, as shown in Figure 6. The results indicate that, compared to the CK group, the fecal microbiome diversity in the LR, L272, and M groups showed an increasing trend, while the Augmentin group exhibited a decreasing trend. However, statistical analysis with the T-test revealed that these inter-group differences were not significant. This suggests that oral administration of *LimosiLactobacillus reuteri* for 21 days did not result in a significant difference in the diversity of the fecal microbiomes of diarrheal mice. On the other hand, antibiotic administration significantly reduced the diversity of the fecal microbiomes (*p* < 0.01). To compare the similarity of species communities between experimental groups, PCA analysis was used for dimensionality reduction and visualization of multiple datasets (Figure 5C). The axes were selected based on the two principal components with the largest variance between samples, and the entire plot explained 99.72% of the variance between groups. As shown in Figure 5C, there is no clear separation among the groups, indicating that the fecal microbiota structure of mice did not show significant differences between experimental groups. Different colors in the plot represent samples from different groups, and the distance between two points reflects the similarity of the fecal microbiota communities between samples [16,17]. Abnormal gut microbiota can impair the intestinal mucosal barrier and exacerbate intestinal inflammation [18]. Probiotics can help mitigate intestinal inflammation by suppressing pro-inflammatory responses and regulating the gut microbiota [19,20].

### 3.7. Analysis of the Fecal Microbiome Community Structure

We analyzed the microbiome communities of the gut in mice from different groups to explore the differences in species abundance between experimental groups, as shown in Figure 6A. At the phylum level, four dominant phyla were detected, with *Firmicutes*, *Bacteroidetes*, and *Proteobacteria* accounting for over 90% of the gut microbiome in each group. Statistical analysis revealed no significant differences in the abundance of *Firmicutes*, *Bacteroidetes*, and *Proteobacteria* among the groups (*p* > 0.05). In the CK group, the average abundances of *Firmicutes*, *Bacteroidetes*, and *Proteobacteria* were 51.39%, 42.19%, and 3.58%, respectively. In comparison to the CK group, the M group saw reductions in *Bacteroidetes* and *Proteobacteria* to 39.67% and 2.2%, respectively, with *Firmicutes* increasing to 54.79%. In the Augmentin group, *Firmicutes* and *Proteobacteria* increased to 58.45% and 6.72%, respectively, while *Bacteroidetes* decreased to 33.35%. In contrast, the LR and L272 groups did not show significant changes in the abundances of *Bacteroidetes* and *Firmicutes* compared to the M group (*p* > 0.05). Notably, in the Augmentin and LR groups, *Bacteroidetes* abundance decreased while *Firmicutes* and *Proteobacteria* abundances increased compared to the M group. Conversely, the L272 group showed a decrease in *Proteobacteria* compared to the M group.

Figure 6B shows that the *Firmicutes*/*Bacteroidetes* (F/B) ratio in the M group is 1.38, while the F/B ratios in the Augmentin and LR groups are 1.75 and 1.59, respectively, indicating an upward trend, although these differences are not significant compared to the M group (*p* = 0.97, *p* = 0.89). The F/B ratio in the L272 group is 1.29, showing a decreasing trend compared to the M group. The relative abundance of species at the phylum level is illustrated in the relative heatmap shown in Figure 6C. According to the similarity dendrogram at the top of the figure, the L272 group and the M group have similar branching patterns and belong to the same branch. They are also closer to the CK group, but more distant from the Augmentin and LR groups. The Augmentin and LR groups are less differentiated from each other and are also closer to the CK group. This suggests that L272 and LR may have functions in maintaining gut microbiota stability in healthy mice.

An analysis of the bacterial communities at the genus level in the fecal samples from each group is shown in Figure 6D. Compared to the model group, the use of probiotics increased the relative abundances of Alistipes, Lactobacillus and Prevotella, while decreasing the relative abundance of Desulfovibrio.

## 4. Discussions

*LimosiLactobacillus reuteri* and *Lacticplantibacillus plantarum* are probiotics naturally present in the gut microbiota of mammals. Research shows that these probiotics play significant roles in inhibiting pathogen infections, preventing and treating inflammation, and regulating gut microbiota. Thus, evaluating the application and functions of these probiotics in pets is valuable for improving their gut microbiota. This study investigated the effects and differences of *LimosiLactobacillus reuteri* and *Lacticplantibacillus plantarum* on the weight, blood inflammation markers, gut morphology, microbiota, and protein expression in the feces of mice with diarrhea induced by *E. coli*, comparing them with antibiotic treatment.

The analysis of weight changes in the gavaged mice indicated that probiotic gavage had a greater impact on weight gain compared to antibiotics, though the exact reasons are unknown. This may be related to the high dose of antibiotics administered. Previous studies have shown that low-dose antibiotics result in a lower average daily weight gain compared to high-dose treatments for *E. coli*-induced diarrhea [21]. The analysis of changes in diarrhea rates and blood inflammation markers in the gavaged mice indicated that the use of probiotics improved diarrhea in *E. coli*-infected mice, but the effect was not as pronounced as that of antibiotics. These results are consistent with Wang et al.’s findings [22], where the use of probiotics improved diarrhea in mice with colitis. Blood inflammation markers in the gavaged mice found that diarrhea led to increased serum levels of sIgA, IL-6, and TNF-α in mice. Yi et al. reported that inflammatory diarrhea raises serum IL-6 and TNF-α levels [23], and Liu found that probiotic treatment reduced sIgA levels and inflammatory factors in diarrhea patients [24]. These findings are consistent with our study, suggesting that diarrhea affects the immune function of mice and that probiotics enhance the intestinal mucosal immune response. The analysis of duodenal sections from gavaged mice revealed that probiotic gavage has certain advantages in maintaining intestinal health. Villus height and crypt depth are important indicators of intestinal health and function [25]. Observing the integrity of villi and crypts can help assess the degree of intestinal damage [26]. Previous studies have shown that diarrhea caused by infections results in thinning of the intestinal wall and significant shortening of villi, which affects nutrient absorption; *LimosiLactobacillus reuteri* has a significant positive impact on small intestinal morphology and plays a crucial role in maintaining the mucosal barrier [27,28]. *Lacticplantibacillus plantarum*, an important intestinal probiotic, protects the intestinal mucosa, enhances intestinal immunity, and alleviates pathological changes. Negotiation research spans many disciplines [29,30]. In this study, after gavaging *LimosiLactobacillus reuteri* and *Lacticplantibacillus plantarum* to *E. coli*-infected diarrhea mice, the intestinal mucosa was more intact and pathological changes were reduced.

The analysis of diarrhea rates and blood inflammation markers in gavaged mice revealed that the MyD88 and NF-kB/p65 signaling pathways in the colon tissues of mice were activated, resulting in noticeable inflammation. Treatment with probiotics LR and L272 led to varying degrees of suppression of MyD88 and NF-kB/p65 signaling pathway proteins, similar to the effect observed with the antibiotic positive control. When the gut microbiota is imbalanced, the immune system is affected, activating MyD88 and NF-kB signaling pathways, which leads to the transcription and translation of downstream pro-inflammatory factor genes and contributes to mucosal inflammation [31]. After adding probiotics, the expression of MyD88 and NF-kB/p65 proteins was inhibited, balancing pro-inflammatory and anti-inflammatory factors and improving mucosal barrier function. Xia’s research on *Lacticplantibacillus plantarum* for alleviating colitis found that this probiotic inhibits the expression of MyD88 and NF-kB/p65 signaling pathways and reduces pro-inflammatory factor TNF-α levels [32], which is consistent with the findings of this study. Combining the trends in inflammation factor changes with alterations in protein expression levels, it was found that in mice gavaged with probiotics, the regulation of protein expression levels helped balance inflammation factor levels and protect gut health.

The analysis of fecal microbiome composition in gavaged mice revealed that *LimosiLactobacillus reuteri* and *Lacticplantibacillus plantarum* did not significantly increase the diversity of the gut microbiota in mice with diarrhea. The reason for this result is not yet clear but may be related to gut damage in the diarrhea-affected mice. Future research could involve feeding healthy mice to continue the investigation. In the LR group, bacteroidetes abundance decreased while Firmicutes and Proteobacteria abundances increased. Hu [33] found that probiotic treatment led to a decrease in Bacteroidetes and an increase in Firmicutes in a study on colitis-induced diarrhea, which is consistent with our results. Previous studies have indicated that inflammatory bowel disease is associated with a higher F/B ratio [34]. In contrast, our study found that while the F/B ratio increased in mice treated with *LimosiLactobacillus reuteri* compared to the model group, it decreased in mice treated with *Lacticplantibacillus plantarum*. This suggests that *Lacticplantibacillus plantarum* may be more effective than *Lactobacillus rhamnosus* in maintaining gut microbiota stability and reducing Proteobacteria abundance. Liang [35] reported that probiotic treatment alleviates diarrhea and increases the relative abundances of Lactobacillus and Prevotella, which is consistent with our findings. The results indicate that administering *LimosiLactobacillus reuteri* and *Lacticplantibacillus plantarum* promotes the abundance of Lactobacillus, which helps establish and maintain gut microbiota balance and alleviate intestinal inflammation.

The application of probiotics offers new strategies for treating diarrhea caused by *E coli*, particularly by reducing the overuse of antibiotics. Probiotics not only alleviate diarrhea but also support gut health and prevent gut microbiota imbalance. Research has shown the role of probiotics in regulating MyD88 and NF-kB p65 signaling pathways, revealing how they help reduce inflammation, enhance gut protection, and promote the restoration of gut health. These findings deepen our understanding of the mechanisms of probiotics, especially in their potential as non-antibiotic treatments to alleviate diarrhea symptoms and reduce antibiotic resistance, with potential applications in both animal and human medicine for diarrhea and intestinal inflammation. Although this study found that *Limosilactobacillus reuteri* and *Lacticplantibacillus plantarum* did not significantly reduce diarrhea rates or increase the gut microbiota richness in mice with diarrhea, mice gavaged with these probiotics showed advantages in weight gain and gut morphology. Additionally, these treatments had a beneficial effect on maintaining gut microbiota stability and increasing the proportion of certain beneficial bacteria.

## 5. Conclusions

We found that these probiotics can improve weight loss caused by diarrhea and provide therapeutic effects against diarrhea induced by *E. coli* infections. This may be related to changes in the gut microbiota structure, reduced inflammatory pathways, and enhanced anti-inflammatory and intestinal protection pathways in mice with *E. coli* K88-induced diarrhea. This study implied that two Lactobacillus strains can alleviate diarrhea and support overall gut health, offering new strategies for the clinical treatment of ETEC-induced diarrhea.

## Figures and Tables

**Table 1:** Diarrhea rate.

Day	Diarrhea Rate, %				
	CK	M	Augmentin	LR	L272
0	—	—	—	—	—
7	—	75	62.5	62.5	62.5
14	—	50	25	25	50
21	—	62.5	12.5	37.5	25

## Data Availability

The data presented in this study are available upon request from the corresponding author.
